# Oxidative Stress and Its Role in Vascular Damage and Atherosclerosis

**DOI:** 10.3390/ijms27021075

**Published:** 2026-01-21

**Authors:** Adela Pozo Giráldez, Adrián Bravo Gómez, Pilar Calmarza, Paula Sienes Bailo, Anita Dayaldasani Khialani, Silvia Montolio Breva, Nerea Sainz-Pastor, Isabel Fort Gallifa

**Affiliations:** 1 Servicio de Bioquímica Clínica y Patología Molecular, Hospital Clínico Universitario de Valencia, 46010 Valencia, Spain; 2 Oxidative Stress Commission, Spanish Society of Laboratory Medicine (SEMEDLAB), 08025 Barcelona, Spain; 3 Servicio de Bioquímica Clínica, Hospital General Universitario Gregorio Marañón, 28007 Madrid, Spain; abravog10@gmail.com; 4 Comisión de Estrés Oxidativo, Sociedad Española de Medicina de Laboratorio (SEMEDLAB), 08025 Barcelona, Spain; mpcalmarza@gmail.com (P.C.); psienesbailo@gmail.com (P.S.B.); anniedayal@gmail.com (A.D.K.); silviamontoliobreva@gmail.com (S.M.B.); nsainz@clinic.cat (N.S.-P.); isafortgal@gmail.com (I.F.G.); 5 Comisión de Lipoproteínas y Enfermedades Cardiovasculares, Sociedad Española de Medicina de Laboratorio (SEMEDLAB), 08025 Barcelona, Spain; 6 Instituto de Investigación Sanitaria Aragón (IIS Aragón), 50009 Zaragoza, Spain; 7 Centro de Investigación en Red en Enfermedades Cardiovasculares (CIBERCV), 28029 Madrid, Spain; 8 Servicio de Bioquímica Clínica, Universitario Miguel Servet, 50009 Zaragoza, Spain; 9 Medicine School, Universidad de Zaragoza, 50009 Zaragoza, Spain; 10 Servicio de Bioquímica Clínica, Hospital Universitario Quirónsalud Zaragoza, 50006 Zaragoza, Spain; 11 UGD de Laboratorio, Hospital Regional Universitario de Málaga, 29010 Málaga, Spain; 12 Hospital Universitari de Tarragona Joan XXIII, 43005 Tarragona, Spain; 13 Servicio de Bioquimica y Genetica Molecular, Centro de Diagnóstico Biomédico (CDB), Hospital Clínic de Barcelona, 08036 Barcelona, Spain; 14 Medicine School, Universitat Rovira i Virgili, 43500 Tarragona, Spain; 15 Institut d’Investigació Sanitària Pere Virgili (IISPV), 43003 Tarragona, Spain; 16 Centre Recerca Biomèdica, 08003 Tarragona, Spain; 17 LaboratoriICS de Tarragona i Terres de l’Ebre, 43003 Tarragona, Spain

**Keywords:** reactive oxygen species (ROS), cardiovascular disease, endothelial dysfunction, vascular inflammation, biomarkers

## Abstract

Oxidative stress (OS) resulting from an imbalance between reactive oxygen species (ROS) generation and antioxidant defenses plays a pivotal role in vascular diseases such as atherosclerosis and hypertension. ROS derived from NADPH oxidase, mitochondria, and xanthine oxidase promote endothelial dysfunction by inducing lipid and protein oxidation, apoptosis, and pro-inflammatory signaling, thereby enhancing smooth muscle proliferation and atherogenesis. This review summarizes the molecular mechanisms linking OS to vascular injury and aims to systematically elucidate the role of OS in vascular diseases, with a specific focus on critiquing the current challenges in translating biomarkers to clinical practice and the emerging trends in personalized antioxidant therapy. Particular attention is given to biomarkers of oxidative stress, including those assessing antioxidant enzyme activity and oxidative damage products, which possess potential for clinical use. Therapeutic strategies targeting OS, including dietary and pharmacological antioxidants, show promise in improving vascular health, although clinical outcomes have been inconsistent and it is necessary to resolve the standardization and validation of these biomarkers, develop precise targeted therapies against specific ROS sources (e.g., NOX inhibitors, mitochondrial antioxidants), and explore personalized clinical trials based on redox stratification. Overall, OS is a central mediator in vascular pathology, and progress in biomarker validation and targeted therapies will be essential to translate current knowledge into effective prevention, diagnosis, and treatment of cardiovascular diseases. Personalized approaches based on accurate redox profiling may enhance efficacy.

## 1. Introduction

Over the past decades, research on oxidative stress (OS) has provided deeper insights into its impact on health and the development of various pathologies. It has been demonstrated that reactive oxygen species (ROS) are implicated in the pathogenesis of numerous neurodegenerative diseases, cancer, and metabolic disorders, and notably in various cardiovascular diseases such as coronary ischemic disease, heart failure, arterial hypertension, and atherosclerosis (AS) [1].

OS is defined as a cellular state where the oxidation–reduction homeostasis is disrupted, indicating an imbalance between ROS production and reactive nitrogen species (RNS) and the body’s capacity to detoxify them. These ROS include free radicals like superoxide, characterized by an unpaired electron in their outer atomic orbital, rendering them highly reactive and unstable. This chemical structure enables them to interact extensively with cellular macromolecules such as carbohydrates, lipids, proteins, and nucleic acids [2].

The human body has natural antioxidant mechanisms to neutralize ROS. However, excessive ROS production or diminished antioxidant capacity can lead to significant cellular damage.

ROS production is essential for maintaining vascular health, but must be tightly controlled to avoid proatherogenic conditions such as inflammation and endothelial dysfunction.

Various environmental factors, including smoking, physical inactivity, unhealthy diet, chronic stress, obesity, viruses, and infectious microorganisms; endogenous biochemical factors such as hypertension, hyperglycemia, hyperhomocysteinemia, high cholesterol, and cholesterol oxidation products, particularly oxysterols; and local hemodynamic abnormalities can all potentially trigger and exacerbate endothelial cell damage, leading to oxidative stress, amplifying vascular inflammation, the transformation of monocytes into macrophages and cell death on the cells of the vascular wall [3]. Oxidative damage to the cell in cardiovascular disease (CVD) causes myocyte dysfunction, which leads to cell death [4]. ROS affect contractile function directly by altering the proteins that are involved in excitation–contraction coupling. Therefore, reduced ROS formation should be employed to prevent and treat cardiovascular disease [5]. The discovery of different biomarkers of oxidative stress warrants further investigation to evaluate CVD [6].

Oxidative stress biomarkers are being developed that may play a promising role in the treatment or diagnosis of diseases where oxidative stress plays a significant role. However, further research is needed to overcome the limitations these markers present, as we will discuss later.

The therapeutic strategies targeting OS modulation include the use of exogenous antioxidants and enhancements in endogenous antioxidant defense [7]; however, we will also consider other new treatments that are not specifically targeted at OS, like the encapsulation of toxic biomolecules such as 7-ketocholesterol (7KC) [8] or the use of synthetic liver X receptor (LXR) agonists that bind to the LXR and can significantly retard the development of atherosclerosis by reducing cholesterol accumulation and inflammatory reactions [9].

The purpose of this review is to demonstrate the relationship between oxidative stress and CVD with emphasis on the following points: oxidative enzymes that cause endothelial dysfunction and oxidative stress biomarkers that are involved in CVD, and the vital role of different antioxidants in lowering free radical level in CVD.

## 2. Genesis of Reactive Oxygen Species

In aerobic metabolism, between 1% and 5% of the oxygen consumed undergoes partial, incomplete reduction, forming reactive intermediate species, known as ROS. These include the radical anion superoxide (O_2_^−^) and hydrogen peroxide (H_2_O_2_), which may or may not have unpaired electrons (radical or non-radical species), but are universally characterized by their high instability and reactivity. Once formed, they can further react to produce more complex species such as peroxynitrite (ONOO−) and hypochlorous acid (HOCl), as well as lipid peroxyl radicals.

These reactive species originate from numerous metabolic processes: prominently, the mitochondrial electron transport chain. Another reactive species, hydroxyl radicals (OH·), which are considered the most cytotoxic of all ROS, can also form and contribute to oxidative damage. The highly reactive nature of ROS enables them to initiate chain reactions to acquire electrons and stabilize.

ROS are generated in various oxidative metabolism reactions, either as reaction intermediates or products thereof. The activation of neutrophils and macrophages leads to the formation of O_2_^·−^ and related species, underpinning their biocidal effects. O_2_^·−^ dismutation yields non-radical hydrogen peroxide or oxygenated water.

**Figure 1:**
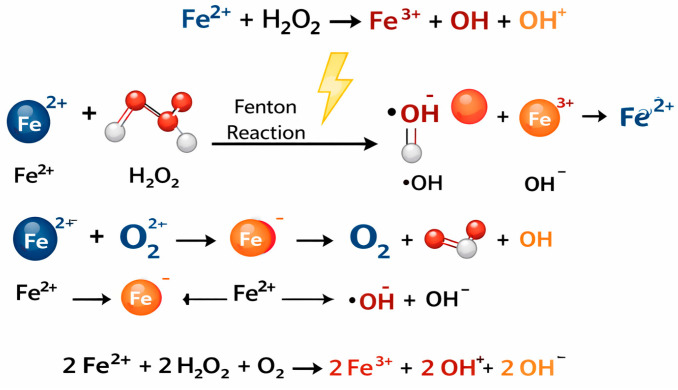
Fenton and Haber–Weiss reactions.

The role of the O_2_^·−^ in generating hydroxyl radicals (OH·) has significant biological implications. Hydroxyl radicals are much more reactive than O_2_^·−^. The formation of hydroxyl radicals occurs through the Fenton reaction. The sum of both reactions is known as the Haber–Weiss reaction [3,10,11,12] (Figure 1).

All types of vascular cells produce ROS, including endothelial cells, smooth muscle cells, fibroblasts, adventitial cells, adipocytes, and phagocytic cells [13,14].

In vascular tissue, the main endogenous sources of ROS generation are as follows:

- Mitochondria are where O_2_^·−^ and H_2_O_2_ are produced from respiratory chain complexes I and II, representing 80% of basal O_2_^·−^ production. Under physiological conditions, the mitochondrial electron transport chain (ETC) leaks small amounts of electrons, but under stress conditions, mitochondrial dysfunction amplifies ROS production, resulting in mitochondrial DNA (mtDNA) damage, impaired oxidative phosphorylation, and the initiation of cell death pathways, including apoptosis and necrosis. Complexes I and III were traditionally considered the most active in ROS generation, but recent studies indicate high activity in complex II as well, with the current understanding suggesting similar activities between complexes I and II, and the highest activity being in complex III [7,15].
- Peroxisomes are multipurpose organelles involved in fatty acid α oxidation, β oxidation of very-long-chain fatty acids, purine catabolism, and the biosynthesis of glycerolipids and bile acids. During the normal catalytic activity of peroxisomes, they produce hydrogen peroxide, a significant ROS [16,17], and play a significant role in the generation of ROS, along with mitochondria. Peroxisome-generated ROS accounts for about 35% of the total intracellular ROS and the main causes of ROS and RNS generation in the peroxisomes are D-amino acid metabolism and peroxisomal-oxidation. They are the sole organelles responsible for the biosynthesis of plasmalogens.
- Nicotinamide adenine dinucleotide phosphate oxidase (NADPH oxidase or NOX) is a multiprotein complex that is crucial for ROS production in various cells and tissues, especially phagocytic cells (neutrophils and macrophages) involved in pathogen elimination and inflammatory processes [18].
- Xanthine oxidase is an enzyme in purine catabolism that catalyzes the oxidation of hypoxanthine to xanthine and then to uric acid, which is also associated with atherosclerosis due to its activation of inflammatory pathways, such as NF-κB, which increase vascular inflammation and exacerbate endothelial damage, thereby promoting the progression of atherosclerosis [13].
- Nitric oxide (NO·) production by endothelial nitric oxide synthase (eNOS) oxidizes L-arginine to citrulline in the presence of calmodulin to form NO·, using BH_4_ as a cofactor. BH_4_ oxidation can lead to non-enzymatic O_2_^·−^ production, limiting eNOS’s ability to produce free NO· in the absence of SOD. This enzyme is the major vascular producer of NO·, but OS can cause uncoupling of eNOS, resulting in O_2_^·−^ production instead of NO· due to BH_4_ oxidation and subsequent deficiency [19,20].
- Inducible nitric oxide synthase (iNOS) predominantly produces NO· from arginine, but BH_4_ deficiency promotes superoxide generation instead of NO·, similarly to eNOS.

**Figure 2:**
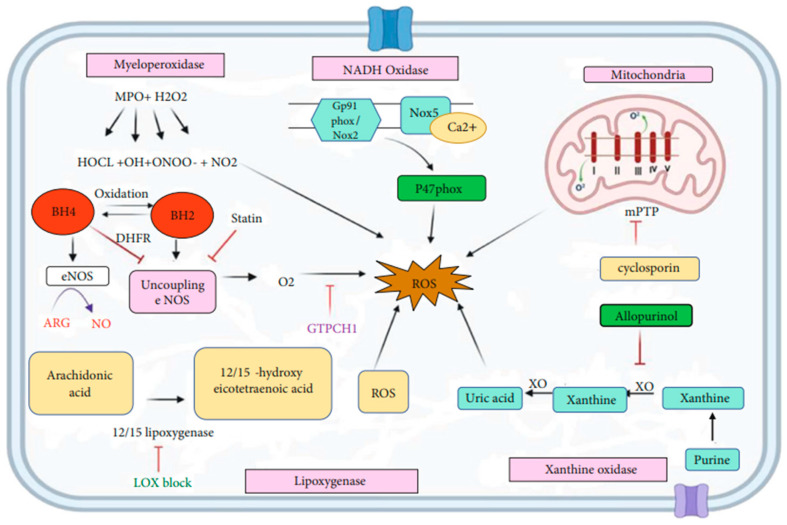
Schematic representation and molecular mechanism of the sources of ROS generation in CVD. Mitochondria is the leading cause of ROS generation; complexes I and III produce superoxides and open mPTP pores in the mitochondrial membrane through ROS release. NOX2/gp91phox generates O_2_^·−^, and other members of the NOX1, NOX4, and NOX5 family are known to be involved in ROS generation. Xanthine oxidase accepts an electron from O_2_^·−^ and O_2_^·−^ while its action can be blocked by allopurinol. Lipoxygenase produces ROS by acting on arachidonic acid into HETE, and LOXBlock-1 blocks this step. When eNOS is uncoupled, it forms O_2_^·−^ instead of NO and helps with ROS production. MPO is the critical oxidative stress biomarker that reacts with H_2_O_2_ and produces various super radicals, which are the primary source of ROS. Adapted from [24].

The mitochondrial ETC is the primary energy source in cardiac cells, driving oxidative-reduction processes that generate ROS. Enzymes such as NADH/NADPH oxidase, xanthine oxidase, lipoxygenase, cyclooxygenase, and nitric oxide synthase catalyze these reactions. Furthermore, fatty acid oxidation in peroxisomes produces hydrogen peroxide: a significant ROS [8,16,21,22]. ROS production can increase in response to factors such as radiation sources, chemical toxins from tobacco, or exposure to environmental toxic agents [1,23]. Figure 2 shows a schematic representation of the main sources of ROS generation in cardiovascular disease.

## 3. Antioxidant Systems and Mechanisms

Just as there are prooxidant molecules, a parallel system of enzymes and molecules known as antioxidants exists. These substances prevent or inhibit the oxidation of other molecules by donating electrons, stabilizing unpaired electrons, or chelating metal ions within their structure. Under physiological conditions, antioxidant concentrations are significantly higher than those of reactive species, ensuring continuous but controlled ROS formation. However, when ROS production becomes excessive and unregulated, endogenous antioxidant enzymes may not be sufficient to neutralize them. Antioxidant systems are classified into enzymatic and non-enzymatic systems, based on their mechanisms of action [1,2,25].

Regarding enzymatic systems, effective antioxidant protection requires the synchronized action of the three main enzymes: superoxide dismutase (SOD), catalase, and glutathione peroxidase (GPx).

- **SOD** catalyzes the dismutation of superoxide radicals (O_2_^·−^) to hydrogen peroxide (H_2_O_2_). Despite being more stable, H_2_O_2_ remains highly reactive. Different molecular variants of SOD have been identified: Cu/Zn-SOD (cytosolic, SOD1), Mn-SOD (mitochondrial, SOD2), and Cu/Zn-SOD (extracellular, SOD3) [2,10,26].
- **Catalases** catalyze the dismutation and peroxidation of H_2_O_2_ into water and oxygen. This is one of the fastest known catalytic activities, making catalases highly effective antioxidants against H_2_O_2_ [27].
- **GPx** removes hydroperoxides and organic peroxides, simultaneously oxidizing its physiological substrate, glutathione (GSH), to oxidized glutathione (GSSG). There are several molecular variants (GPx1 to GPx5), both selenium-dependent (tetrameric) and selenium-independent (dimeric) [28].

In general, a tight relationship exists between the activity levels of these enzymes and the concentration of various biometals, usually serving as cofactors of these enzymes. For example, copper (Cu^2+^) and zinc (Zn^2+^) ions, in particular, have a great impact on the activity of cytoplasmic SOD, whereas manganese (Mn^2+^) is a metal that is essential to the function of the mitochondrial type of this enzyme (mSOD). Iron ions (Fe^3+^) are an integral component of catalase and almost no oxidant enzymatic action can be managed without specific ion equilibrium.

In addition to these enzymes, other low molecular weight molecules exhibit non-enzymatic antioxidant activity. Notably, ascorbic acid (vitamin C) reduces ROS to water, with its oxidized species being non-reactive, and Vitamin E (alpha-tocopherol) degrades peroxides to hydroperoxides, which can then be further degraded by reduced glutathione (GSH), one of the most effective endogenous antioxidants. GSH detoxifies xenobiotics via GSH-transferase, maintains the reduced state of many proteins, and neutralizes OH·. Flavonoids also degrade peroxides to non-reactive forms, and carotenoids function as antioxidants due to their system of conjugated double bonds [29,30,31].

Furthermore, proteins that are capable of metal coordination, such as ferritin, transferrin, albumin, or ceruloplasmin and molecules like carotenoids, vitamins, flavonoids, and enzymes such as SOD and other metallothioneins, remove excess ROS through enzymatic or antioxidant compound action [31,32]. Heat shock proteins (HSPs) constitute another defense mechanism by safeguarding cellular proteins from OS-induced damage, through the facilitation of proper folding and repair of damaged proteins [33].

Continuous research in this domain remains essential for the development of novel therapeutic strategies aimed at augmenting endogenous antioxidant defenses and mitigating the deleterious effects of oxidative stress on human health [3].

## 4. Oxidative Stress and Cardiovascular Disease Pathogenesis

Cardiovascular disease (CVD) is the most common cause of death globally and the origin in the majority of cases of CVD is atherosclerosis, a chronic arterial disease characterized by accumulation of inflammatory cells and lipids in the blood vessel wall.

The activation of the immune system due to disrupted redox homeostasis induces an inflammatory state that creates a vicious cycle in which chronic oxidative stress and inflammation feed off of each other.

Oxidative stress has been shown to play a major role in the pathogenesis of atherosclerosis, because apart from the production of oxidized lipoproteins, it also causes direct damage to the cellular and nuclear membranes and interacts with endogenous vasoactive mediators in endothelial cells.

Furthermore, in recent years, special attention has been paid to the transcription factor Nrf2 and its downstream-regulated protein heme oxygenase-1 as protectors against atherosclerotic injury, and a recent study suggests that NOX5, by mechanisms linked to increased intracellular calcium, is the key to early lysophosphatidylcholine-induced endothelial oxidative stress and the pro-inflammatory processes of atherosclerosis.

This process is initiated with endothelial cell injury and dysfunction [34] and this damage increases vascular permeability, facilitating positively charged lipoproteins to penetrate and accumulate in the negatively charged, proteoglycan-rich subendothelial region and undergo oxidative modification. Reactive oxygen species (ROS) oxidize LDL into oxLDL that triggers T cell-mediated autoimmune responses and endothelial injury.

At this phase, expression levels of Vascular cell adhesion molecule 1 (VCAM-1), Intercellular adhesion molecule 1 (ICAM-1), Monocyte chemoattractant protein-1 (MCP-1), P-selectin, and E-selectin are upregulated, they guide monocytes to transverse the endothelium, and they are transformed into macrophages, which—along with vascular smooth muscle cells—internalize oxLDL via scavenger receptors, transforming them into foam cells filled with substantial cholesterol esters. Subsequently, the vascular smooth muscle cells (VSMCs) migrate from the media to the intima and produce extracellular matrix components like collagen, elastin, and proteoglycans [35]. These processes contribute to the formation of a fibrous cap and thickening of the vessel wall, imparting stability to the plaque and resulting in luminal narrowing [36].

Thinning of fibrous caps coupled with the expansion of necrotic core zones may lead to plaque rupture, exposing prothrombotic materials and triggering platelet activation, thrombus formation, and ultimately local blood flow interruption. Furthermore, we now know that ferroptosis also damages endothelial cells and macrophages and plays a crucial role in the development of atherosclerosis, which also contributes to plaque instability and disease progression [37]. It represents a form of non-apoptotic programmed cell death that is characterized by the iron-dependent accumulation of lethal lipid reactive oxygen species (ROS) and the peroxidation of membrane polyunsaturated fatty acid phospholipids.

On the other hand, 7-Ketocholesterol (7KC), which is one of the most important oxysterols produced by the autoxidation of cholesterol, is also a toxic and highly proinflammatory molecule that initiates an inflammatory response in endothelial cells (EC), which facilitates the recruitment of immune cells, including monocytes, which differentiate into macrophages.

Due to subtle but important structural differences between 7KC and cholesterol, excessive 7KC leads to the transformation of macrophages into foam cells: one of the primary components of atherosclerotic plaque [38]. Through dysfunction of their lysosomal system, foam cells become engorged with lipids and are incapable of participating in reverse cholesterol transport (RCT). During disease progression, 7KC presence leads to increased oxidative stress, intracellular lipid accumulation, and the inability to phagocytose, leading to an increase in apoptosis in macrophages and a contribution to plaque vulnerability and instability [38].

**Figure 3:**
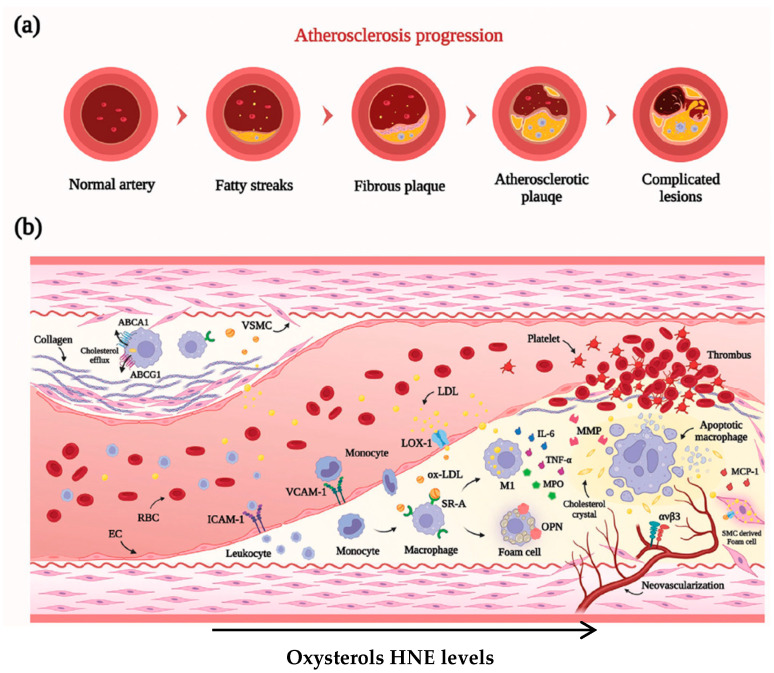
Schematic illustration of the development of atherosclerosis and pathological characteristics of different types of atherosclerotic plaques. (**a**) The four stages of pathological progression of AS. (**b**) The vulnerable plaque (**bottom right**) has a high risk for rupture and thrombosis and is composed of a lipid-rich necrotic core, inflammatory cells and cytokines, thin fibrous cap, and neovascularization, compared with stable plaque (**top left**) [39].

Accumulation of oxysterols and HNE contributes to plaque progression and recent studies show that plasmalogens are also modified in the plaques, which are an important site of oxidative stress. Continuous LDL oxidation and lipid release from apoptotic foam cells produce increasing levels of oxysterols and HNE, which contribute to all stages of atheroma formation. These oxidized lipids sustain the inflammatory process and endothelial activation, the formation of foam cells, the migration of SMCs from the tunica media toward the intima, and degradation of the fibrous cap, favoring plaque rupture and thrombus formation, as shown in Figure 3.

## 5. Possible Mechanisms of Genesis of Cardiovascular Diseases

### 5.1. Endothelial Dysfunction

The endothelium, the inner layer of blood vessels, plays a key role in homeostasis. In response to various stimuli, it releases vasodilatory, anticoagulant, and anti-inflammatory factors like NO, vasoconstrictive and pro-aggregatory factors such as endothelin-1 (ET-1) and Angiotensin-II [40,41].

The effects of ROS on cardiovascular function depend on their concentrations and antioxidant production [42]. Elevated thiol levels protect cells from OS-induced death, while depletion leads to disease states [43,44].

NADPH oxidase, found in endothelial cells, vascular smooth muscle cells, and fibroblasts (NOX1, NOX2, NOX4, and NOX5), is a primary source of O_2_^·−^ in the vessel wall. Physiologically, it has low activity but is upregulated by angiotensin II, ET-1, and urotensin II, increasing ROS production and contributing to cardiovascular disease [7,9,19,45].

NO· acts as a crucial paracrine regulator of vascular tone, exerting potent vasodilatory effects by increasing cGMP levels through guanylate cyclase activation. Reduced NO· availability leads to endothelial dysfunction and cardiovascular diseases. O_2_^·−^ generated from these conditions reacts with NO· to form peroxynitrite, exacerbating endothelial barrier dysfunction and promoting LDL accumulation and leukocyte adhesion in arterial walls, triggering inflammation [46]. NADPH oxidase-driven O_2_^·−^ production further reduces NO· levels by forming ONOO−, disrupting endothelial function and eNOS activity by oxidizing BH_4_. This shift favors O_2_^·−^ production over NO·, contributing to hypertension, atherosclerosis, and other cardiovascular disorders. Xanthine oxidase, activated by NADPH oxidase, amplifies O_2_^·−^ production, exacerbating OS. Myeloperoxidase catalyzes hypochlorite production, intensifying inflammation and arterial damage and promoting atherosclerotic plaque formation and cardiovascular disease progression [47].

The alterations caused by OS disrupt normal endothelial function, leading to an imbalance between vasoconstrictive and vasodilatory substances and these alterations affect the anticoagulant and proinflammatory properties of the endothelium [9,23,48].

### 5.2. Mitochondrial Dysfunction

Mitochondrial ROS production is considered to be the most significant source of free radicals under physiological conditions, due to the ETC located in the inner mitochondrial membrane. These radicals are continuously generated during oxidative phosphorylation and ATP production [49].

Mitochondrial dysfunction in cardiovascular diseases can manifest in several ways:

1. **Reduced ATP production**: Impaired mitochondrial function leads to decreased ATP production, reducing myocardial contractility and overall cardiac function [50,51].
2. **Increased ROS production**: Continuous production of O_2_^·−^ and other ROS in mitochondria increase the oxidative damage to biomolecules, particularly nuclear and mitochondrial DNA (mtDNA). mtDNA instability is crucial in mitochondrial dysfunction, leading to mutagenic or cytotoxic effects and subsequent mutations after DNA replication. The accumulation of mtDNA mutations contributes to tissue function loss, including in the heart [50,51,52,53]. Animal models have confirmed this increased mtDNA instability [54].
3. **Calcium homeostasis imbalance**: Mitochondria regulate the intracellular calcium concentration, which is essential for cardiac contraction, and mitochondrial dysfunction disrupts calcium homeostasis, affecting cardiac contractility and potentially contributing to arrhythmias [55].
4. **Apoptosis induction**: Mitochondrial dysfunction can trigger the intrinsic apoptosis pathway, regulated by the release of pro-apoptotic proteins from mitochondria to the cytosol. Excessive apoptosis of cardiac cells reduces cardiac muscle mass, leading to various pathologies [56,57].
5. **Inflammation**: Dysfunctional mitochondria can activate inflammatory responses in cardiac and other cardiovascular cells, contributing to the progression of diseases, such as atherosclerosis and ischemic heart disease [57].

### 5.3. Oxidative Modifications Induced by ROS

ROS cause cellular damage to various biomolecules. The primary cytotoxic effects of oxidative/nitrosative stress result from the interaction of oxygen and nitrogen radicals with the cell membrane, lipids, proteins, and nucleic acids [58].

**Lipid peroxidation and LDL oxidation:** Oxidation of low-density lipoproteins (LDL) is a free-radical-mediated process, causing significant structural changes. The initial event is the peroxidation of polyunsaturated fatty acids (PUFA) in LDL particles. This peroxidation alters membrane properties, potentially inactivating membrane receptors or enzymes, and affecting normal cellular function. Oxidized LDL (oxLDL) stimulate vascular ROS formation, creating a vicious cycle. Myeloperoxidase, expressed in macrophages within atherosclerotic lesions, primarily catalyzes the complete oxidation of LDL, along with glycosylases. The number, composition, and oxidation susceptibility of LDL particles also play crucial roles [59,60].

**Stages of lipid peroxidation:**

1. **Initiation:** ROS extract a hydrogen ion (H+) from a PUFA double bond, forming conjugated dienes (CD). Antioxidants in LDL particles initially halt this oxidation.
2. **Amplification:** Once antioxidants are depleted, another H+ is abstracted by a peroxyl radical (LOO·) from a PUFA, forming lipid hydroperoxides. This increases LDL’s negative charge, leading to macrophage recognition via scavenger receptors in arterial intima.
3. **Decomposition:** Double bonds break, forming aldehydes like malondialdehyde (MDA), 4-hydroxynonenal (HNE), and hexanal, which react with proteins and nucleic acids, leading to cytotoxic and mutagenic effects and playing a pathogenic role in various diseases [59,60,61,62].

**Endothelial damage and inflammation:** Endothelial lesions facilitate LDL entry into the vascular intima, where oxidation triggers an inflammatory response, causing cytokine expression (e.g., IL-4, IL-1β, TNF-α) and adhesion proteins (e.g., VCAM-1, ICAM-1, P-selectin). These allow for monocyte migration and transformation into macrophages and foam cells after phagocytosing oxLDL, promoting atheroma plaque formation and its physiological consequences [63].

**Lipid peroxidation amplification:** Lipid peroxidation rapidly propagates through plasma membranes, and the mutagenic potential of its products makes this mechanism significant in OS toxicity [63]. This propagation or amplification can continue or decrease, depending on the antioxidant defense’s efficiency.

**High-density lipoproteins (HDL)** also undergo oxidative modifications that impair their anti-atherogenic properties. Oxidation by agents like copper or acrolein reduces HDL’s cholesterol transport activity and cellular cholesterol efflux via the ATP Binding Cassette A1 (ABCA1) transporter. Specifically, oxidation by malondialdehyde and myeloperoxidase at the methionine 112 residue of apolipoprotein A1 (Apo A1) diminishes HDL’s capacity to inactivate lipid hydroperoxides. Reactive carbonyl-induced covalent modifications further impair HDL-mediated cholesterol efflux [64,65,66,67,68].

## 6. Consequences of Oxidative Stress: Atherosclerosis, Hypertension, Heart Failure, Ischemic Heart Disease, and Diabetic Cardiomyopathy

Atherosclerosis begins with the infiltration of oxLDL into the arterial wall through the endothelium.

This infiltration triggers inflammatory processes including ROS production by macrophages, which, along with oxLDL, contribute to the formation of atheromatous plaques and foam cells, exacerbating the vascular damage [43,69,70].

Regarding hypertension, ROS reduce nitric oxide (NO) bioavailability by reacting with NO· to form peroxynitrite, a potent oxidant, resulting in impaired endothelium-dependent vasodilation. This imbalance induces the expression of vascular adhesion molecules, accelerating atherosclerosis, and ROS such as O_2_^·−^ reduce the availability of NO, which is essential for arterial smooth muscle relaxation [71]. Additionally, redox-sensitive pathways promote vascular smooth muscle cell proliferation and extracellular matrix remodeling, contributing to increased arterial stiffness and elevated systemic vascular resistance.

Furthermore, OS is associated with structural changes in resistance arterioles and premature endothelial cell senescence [69,72,73].

Regarding heart failure (HF), persistent ROS overproduction induces mitochondrial DNA damage, disrupts ATP production, impairs calcium handling, and promotes cardiomyocyte apoptosis. ROS activate various kinases and transcription factors that promote myocardial growth, matrix reorganization, and cellular dysfunction [43,74]. They induce hypertrophy in ventricular myocytes and affect the extracellular matrix by stimulating cardiac fibroblast proliferation and matrix metalloproteinase activation, which are relevant in fibrosis and matrix remodeling. Several studies on animal models have demonstrated that the administration of antioxidants can prevent pathological phenomena associated with heart failure, such as myocyte hypertrophy, apoptosis, ischemia, and reperfusion [75].

There are also other effects of free radicals in the pathogenesis of HF, through the xanthine oxidase pathway, which include endothelial dysfunction and myocardial injury. Increased ROS production can lead to decreased NO bioavailability and cytokine-mediated myocardial contractile dysfunction, which inactivates the sarcoplasmic Ca^2+^-ATPase, thereby altering calcium homeostasis [76,77].

On the other hand, diabetes mellitus (DM) is a primary risk factor for the development of atherosclerosis, which is closely associated with increased OS. Hyperglycemia, which is characteristic of both type 1 and type 2 DM, triggers ROS through various mechanisms, including glucose auto-oxidation and non-enzymatic glycation of proteins. Pathological processes exacerbated in DM, such as the increased production of advanced glycation end-products (AGEs) and their interaction with endothelial and smooth muscle cells that enhance ROS production and activate nuclear factor kappa B (NF-κB), contribute to increased inflammation and thrombosis in the vascular endothelium. Consequently, endothelial dysfunction is observed, characterized by reduced NO bioavailability and calcium homeostasis alterations, leading to increased arterial stiffness and impaired vascular reactivity. These phenomena promote the progression of atherosclerosis and associated cardiovascular complications, underscoring the importance of glycemic control in mitigating these pathological effects [78,79].

## 7. Parameters of OS in Cardiovascular Disease

OS biomarkers are valuable tools for the development of new preventive, diagnostic, and therapeutic strategies aimed at preventing or delaying the onset of pathologies such as arteriosclerosis and cardiovascular diseases. Their use allows for the identification of oxidative damage in cells and tissues, and the monitoring of responses to antioxidant treatments and other therapeutic interventions [24,79,80,81,82,83,84,85,86,87,88,89,90,91,92,93,94,95,96]. Recent integrative reviews highlight the clinical potential of multi-marker panels and redox ratios for cardiovascular risk stratification [80].

The first studies on OS biomarkers, dating back to the 1980s, identified several direct or indirect indicators of reactive oxygen and nitrogen species (ROS/RNS) activity in vivo. These biomarkers should ideally do the following: (i) represent oxidative damage associated with disease onset or progression, (ii) be quantifiable in target tissues or plasma, (iii) be specific and reproducible, and (iv) remain stable during sampling and processing [81].

However, clinical trials have often failed due to inadequate biomarker selection or lack of methodological standardization. Measuring the antioxidant compound itself in plasma, for instance, does not reflect the oxidative status [82]. Because of the complexity of OS-related diseases, no single biomarker can capture the redox state or predict clinical outcomes.

Direct detection techniques, such as electron paramagnetic resonance (EPR) and spin trapping, remain the gold standard for identifying free radicals but are technically demanding and rarely applicable to large cohorts. Therefore, most clinical studies rely on indirect markers determined by gasometric, spectrophotometric, immunoenzymatic (ELISA), or chromatographic techniques [83].

Methods of quantification for lipid peroxidation include measurement of peroxide formation, oxygen consumption, and formation of conjugated dienes by spectroscopic methods [84,85].

Below are some of these biomarkers, which are potentially useful for studying oxidative stress processes associated with cardiovascular pathology.

- **Malondialdehyde (MDA):** This is formed from the breakdown of lipids containing polyunsaturated fatty acid, derived from arachidonic acid metabolism. Tissue damage can elevate MDA concentrations, which can react with lysine residues, leading to protein alterations that are associated with cardiovascular diseases such as arteriosclerosis or acute myocardial infarction [24]. The determination of this marker can be performed using colorimetric, immunoenzymatic (ELISA), or HPLC techniques [83]. Although easy to measure, concerns remain regarding the specificity and reproducibility of MDA assays. It is influenced by diet and sample handling.
- **Acrolein and 4-Hydroxy-2-nonenal (HNE):** Both of these are reactive aldehydes derived from lipid oxidation by ROS/RNS, forming adducts with proteins and nucleic acids and promoting cytotoxicity and apoptosis [84]. They are determined by HPLC or GC-MS [83]. They present a short half-life and preanalytical instability.
- **F2 Isoprostanes:** These are non-enzymatic products of arachidonic acid oxidation that are detectable in plasma and urine, representing one of the most reliable lipid peroxidation markers in vivo [97]. There is increasing evidence focusing on the association of increased F2-IsoP with CVD risk factors, but there is limited research linking this biomarker to clinical outcomes. The ability of F2-IsoP in promoting vasoconstriction and inflammation further supports its potential involvement in the development of hypertension and its ability to retain its stability in urine allowed it to assess oxidative stress status in vivo accurately [86]. Measurement of urinary or plasma 8-iso-PGF2α serves as a reliable and specific indicator of lipid peroxidation and oxidative injury. Its determination can be carried out using immunoenzymatic (ELISA) or GC-MS, LC-MS/MS techniques [83].
- **Myeloperoxidase (MPO):** This is a heme-containing peroxidase released by activated neutrophils and monocytes that catalyzes the production of reactive oxidants such as hypochlorous acid during inflammation and correlates with plaque vulnerability. Quantified by ELISA, it is sensitive to preanalytical factors (heparinization, temperature). Results depend on preanalytical handling. Elevated plasma MPO levels are associated with endothelial dysfunction, plaque instability, and an increased risk of myocardial infarction and heart failure. It is the most efficient marker in detecting AMI patients and improves the risk classification of NSTMI. MPO serves both as a biomarker and as a potential therapeutic target in CVDs.
- **Oxidized LDL (oxLDL):** In the presence of hydrogen peroxidase, MPO can induce oxidative modification on LDL and release oxidized LDL (ox-LDL) [87]. OxLDL is a central player in the development of aterosclerosis, triggering endothelial dysfunction and plaque instability. Higher levels of ox-LDL were associated with patients with very early CAD, compared to a control group. Circulating oxLDL levels correlate with the extent of coronary artery disease and are predictive of adverse cardiovascular events. Ox LDL also promotes a pro-inflammatory state by activating scavenger receptors and innate immune responses. It is detected by using monoclonal antibodies against oxidized epitopes through ELISA methods, but the assays lack standardization.
- **Malondialdehyde-modified low-density lipoprotein (MDA-LDL)** is similar to ox-LDL and represents one of the major products of lipid peroxidation [88]. MDA-LDL was able to reflect the presence of vulnerable plaque and independently predict adverse cardiovascular events in ACS patients with successful non-surgical percutaneous coronary intervention.
- **Thiols:** Total thiol (TTL) appears early in CVD onset and represents the redox control status of vascular systems [89]. They are early indicators of redox imbalance and their increase correlates with cardiovascular events in middle-aged individuals (45–60 years), although more studies are needed to establish a clear relationship with acute myocardial infarction.
- **Oxysteroles:** In order to degrade cholesterol to more polar compounds, an oxygen function, such as a hydroxyl, epoxide, or ketone group, is introduced into the sterol nucleus or side chain. Oxysterols are formed either by autoxidation, enzymatically, or by both mechanisms and, due to the additional oxygen function, are readily able to cross lipophilic membranes, unlike cholesterol. In particular, the oxysterols 7alfa hydroxycholesterol (7αOH), 7 beta hydroxycholesterol (7b-OH) and 7 ketocholesterol (7-K) have been demonstrated to induce a clear inflammatory phenotype in human endothelial cells, and 5,6-secosterol is a potent inhibitor of endothelial-dependent arterial relaxation. Through processes like oxidative stress, their accumulation causes cytotoxicity, which in turn results in cell death. In atherosclerosis, they have a role in plaque instability, foam cell production, and endothelial dysfunction. Furthermore, by influencing inflammatory signaling, reactive oxygen species (ROS) generation, and lipid peroxidation, they increase cellular damage and speed up the etiology of disease. OxLDL contains many oxysterols, and 7KC accounts for 30% of the total cholesterol in oxLDL. Some studies also indicate that the plasma 7b-OH cholesterol level can be used as a biomarker for detecting carotid and coronary artery disease, but further clinical studies are needed to evaluate the potential of oxysterols for use as biomarkers for plaque vulnerability and instability. They are measured by GC-MS.
- **Plasmalogens:** These are a specific glycerophospholipid class, containing a vinyl ether moiety at the sn-1-position of the glycerol backbone. Plasma plasmalogens may reflect the systemic functional activity of peroxisomes and serve as potential biomarkers of oxidative stress. Jalil et al. [98] revealed that macrophage phospholipid metabolism is regulated by the key enzymes LPCAT3 and ELOVL5. A depletion of lipid molecules generated by the LPCAT3-ELOVL5 axis, especially of the content of arachidonic-acid of plasmalogens, could increase macrophages sensitivity to cytotoxic oxysterols, such as 7 ketocholeterol. The analysis of human atheroma plaques also revealed that plasmalogens generated through the LPCAT3-ELOVL5 pathway, especially arachidonic-acid-enriched plasmalogens, correlate with a more favorable plaque profile and show a negative association with oxysterol levels [98].
- **Glutathione and S-glutathionylated proteins:** Since blood concentrations of glutathione reflect the status of glutathione in other less accessible tissues, measurements of reduced glutathione (GSH) and glutathione disulfide (GSSG) in blood are considered essential as an index of the GSH status throughout the body and are a useful indicator of oxidative stress status in humans. The GSH/GSSG ratio reflects a dynamic equilibrium and is not influenced by external factors or specific damage conditions in the body (as MDA might be, which depends on lipid peroxidation). This type of measurement can provide more functional data on the cellular health status over time [91].
- **Antioxidant enzyme activities**—superoxide dismutase, catalase, glutathione peroxidase—and their ratios to oxidative products provide integrative information on redox homeostasis [24,89,94,96,97,98]. Non-enzymatic antioxidants (ascorbate, glutathione, trace elements such as zinc, selenium, manganese) can be measured calorimetrically, by atomic absorption or GC-MS [83].

Table 1 shows the main oxidative stress biomarkers studied in cardiovascular disease.

## 8. Limitations and Future Directions

Unfortunately, reference values for all these types of markers are still unknown. Factors such as age, gender, nutritional status, or health condition can affect these biomarkers, as well as the lack of standardization in measurement techniques, as there is no widely accepted consensus on the most appropriate analytical method for determining these markers. Different analysis techniques may generate results that are not comparable with one another, making clinical interpretation more difficult. Although these biomarkers may indicate cellular damage, it is not always clear to what extent they reflect the progression of specific diseases. Furthermore, despite the fact that in diseases like cardiovascular conditions, oxidative stress is just one of many contributing factors, other additional factors complicate the interpretation of these markers, as specific indicators of pathology. Additionally, it should be emphasized that many of these oxidative stress biomarkers are highly reactive and can break down quickly in uncontrolled conditions. This means that proper sample handling and storage are essential to obtain reliable results. For all these reasons, currently, the applicability of oxidative stress biomarkers is limited to a few laboratories: specifically, research laboratories, which employ their own reference values based on their study population [83,92,93].

Furthermore, the clinical translation of oxidative biomarkers remains limited. The lack of standardized reference ranges, variability in analytical methods, and preanalytical instability hinder comparability and reproducibility [83,92,93]. Age, sex, nutritional status, and comorbidities further modulate biomarker levels.

Future efforts should focus on establishing validated analytical methods, population-based reference intervals, and multi-biomarker panels that integrate lipid, protein, and nucleic acid oxidation products to improve diagnostic accuracy in cardiovascular disease.

## 9. Nutritional and Therapeutic Strategies to Reduce Oxidative Stress

During the 1990s and early 2000s, antioxidant therapies were primarily focused on the administration of antioxidant vitamins—particularly vitamin E, vitamin C, and β-carotene—with the aim of counteracting lipid oxidation and preventing atherogenesis.

Flavonoids, key bioactive components in the diet, exhibit antioxidant, anti-inflammatory, and cardioprotective properties. They contribute to lipid and blood pressure, myocardial ischemia, and arrhythmias reduction. Epidemiological studies have established an inverse correlation between flavonoid consumption and cardiovascular mortality. These compounds act through multiple mechanisms including the NO-guanylate cyclase pathway, endothelium-derived hyperpolarizing factors, and ET-1 to protect endothelial cells from apoptosis. They also reduce circulating oxidized LDL concentrations and aortic stiffness, thus improving endothelial function. Clinically, foods such as green tea, dark chocolate, grape anthocyanins, and quercetin from onions have shown improvements in endothelial function in hypertensive and ischemic heart disease patients [76,99,100,101,102,103,104,105].

Various nutritional compounds including vitamin C [106,107]; antioxidant-rich nuts containing selenium, zinc, vitamins A, E, and tocopherols [106,108]; probiotics [109]; and minerals like potassium, calcium, and magnesium demonstrate significant positive effects on cardiovascular health [106]. Specifically, Ginkgo Biloba and curcuminoids from turmeric have been studied for their ability to activate the SIRT1 axis, reduce inflammation, preserve cardiomyocytes, increase NO bioavailability, and decrease cardiac OS and myocardial apoptosis [110,111,112,113]. Additionally, research suggests that diets enriched with extra virgin olive oil can reduce the risk of acute myocardial infarction, stroke, and cardiovascular-related mortality by 30% [114]. These dietary strategies contribute to preventing the uptake of oxidized LDL by arterial wall cells, promoting vasodilation, improving endothelial function, reducing inflammation, and mitigating OS. Antioxidants, both natural and synthetic, play a crucial role in early prevention of cardiovascular diseases and are effective when used at optimal concentrations. Compounds with polyphenolic structures and hydroxyl groups (OH) are particularly effective due to their antioxidant activity [90].

However, the Heart Outcomes Prevention Evaluation (HOPE), the Gruppo Italiano per lo Studio della Sopravvivenza nell’Infarto Miocardico (GISSI), and the Heart Protection Study (HPS), did not demonstrate reductions in the incidence of myocardial infarction, stroke, or cardiovascular mortality with vitamin E or β-carotene supplementation. Despite many studies that have tested the effects of antioxidants on oxidative stress in patients with CAD, the literature still lacks an updated and comprehensive systematic review. In this sense, a study was conducted in 2025 [115] to identify the effects of administering exogenous antioxidants on OS levels among adult patients with CAD. In this study, a systematic review searched PubMed, Medline, and CINAHL for randomized controlled trials (RCTs) published between January 2013 and May 2025, which examined antioxidants to lower OS in adult participants with CAD. Among 2338 studies reviewed, 15 RCTs met the inclusion criteria and of the 15 RCTs, 9 reported on supplemental antioxidants (i.e., L-carnitine and melatonin) and 2 reported on dietary antioxidants (Khorasan wheat diet and wine) that were effective at lowering OS (*p* < 0.05). The reviewed RTCs provide evidence that antioxidants may lower OS in patients with CAD but the utility of this conclusion is limited by the heterogeneity in methodologies between studies, which indicates that further research is needed with standardized interventions and outcomes.

On the other hand, the nuclear factor erythroid 2-related factor 2 (Nrf2) pathway is a critical defense mechanism against oxidative stress, helping to protect cardiac function under pathological conditions, and oxidative stress is the key pathological feature implicated in the development of dyslipidemia-associated cardiovascular complications [116]. The activation of Nrf2, which is a transcription factor that belongs to a family of conserved amino acids, plays a critical role in maintaining redox homeostasis by promoting the expression of enzymes like superoxide dismutase (SOD), catalase (CAT), and glutathione peroxidase (GPx), which work to neutralize reactive oxygen species (ROS) and protect myocardial cells from oxidative damage [117,118].

While statins and metformin are primarily known for their lipid- and glucose-lowering effects, emerging evidence suggests that they also influence Nrf2 activation and beyond their primary lipid-lowering effects, statins have been shown to exert pleiotropic effects that modulate oxidative stress, potentially through Nrf2 activation [119]. Similarly, metformin, the first-line treatment for type 2 diabetes (T2D), has been reported to influence Nrf2 pathway activation through its effects on mitochondrial function and AMP-activated protein kinase (AMPK) signaling [120]. Therefore, activating Nrf2 represents a promising therapeutic strategy for managing oxidative stress and improving outcomes in patients with dyslipidemia-associated cardiovascular complications. These therapies, along with the use of NADPH oxidase inhibitors and mitochondrial-directed antioxidants, represents a promising next-generation approach designed to overcome the shortcomings of broad-spectrum antioxidant supplementation.

The NO pathway also represents a promising therapeutic target in both stable and advanced heart failure. Modulating this pathway could improve outcomes, particularly in complex populations with multiple comorbidities, and various studies have also demonstrated that angiotensin-converting enzyme inhibitors (ACEIs) such as Captopril and calcium channel blockers possess antioxidant properties. Their vascular protective effect is mediated by reducing OS through thiol groups, positively regulating eNOS expression, and inhibiting NADPH oxidase activity, thus restoring vascular defense activity via endogenous antioxidants like extracellular SOD [43,99].

Combination therapies with renin–angiotensin–aldosterone system (RAAS) inhibitors in acute myocardial infarction patients have shown reduced vascular OS and myocardial damage, proving to be more effective in preventing the harmful effects of vascular OS compared to vitamin supplementation.

Furthermore, some publications discuss inhibition of ASK1-signalosome, a key protein complex regulating OS that links ROS generation to the signaling pathways involved in aging and cardiovascular diseases. Thioredoxin (Trx) acts by keeping ASK1-signalosome inactive when reduced and is thus considered a potential cardioprotective therapy against OS [47,99]. Further investigation into OS biomarkers is crucial for personalized treatments and improved outcomes in vascular diseases. Standardizing these biomarkers will also aid in assessing disease severity and treatment efficacy. Advances in omics technologies, including redox proteomics, metabolomics, and transcriptomics, offer new opportunities for patient phenotyping and biomarker discovery, which could facilitate personalized interventions.

### 9.1. The Mediterranean Diet as a Model of Comprehensive Antioxidant Intervention

In 2004, the American Heart Association (AHA) conducted a comprehensive review of more than twenty randomized controlled trials and concluded that there was no solid evidence supporting the routine use of antioxidant supplements for the prevention or treatment of cardiovascular disease [121]. Integrating lifestyle interventions with pharmacological treatments may yield more sustainable benefits by addressing both the causes and consequences of oxidative imbalance.

Dietary patterns of populations with long life-spans, like the traditional Mediterranean diet and the Okinawa Island diet, provide the basis to recommend plant foods like vegetables, legumes, fruits, non-tropical vegetable oils as a basic fat, light meat (e.g., poultry) of moderate amounts, plenty of fish, and moderate beverage intakes of wine, coffee, and tea.

The Mediterranean diet represents the most scientifically supported nutritional paradigm for cardiovascular prevention and antioxidant protection. Its efficacy is grounded in the synergistic interaction of plant-derived bioactive compounds, including polyphenols, tocopherols, carotenoids, and mono- and polyunsaturated fatty acids, which act across multiple metabolic, redox, and inflammatory pathways.

The PREDIMED trial (Prevención con Dieta Mediterránea), published by Estruch et al. (2018) [122] in the New England Journal of Medicine, stands as one of the largest primary prevention studies on cardiovascular disease (CVD). This randomized trial enrolled 7447 Spanish participants who were at high cardiovascular risk but without prior CVD, assigning them to one of three dietary interventions: (a) a Mediterranean diet supplemented with extra-virgin olive oil, (b) a Mediterranean diet supplemented with mixed nuts, or (c) a control low-fat diet. After a median follow-up of 4.8 years, the groups assigned to a Mediterranean diet exhibited a 30% relative reduction in major cardiovascular events (myocardial infarction, stroke, or cardiovascular death) compared with the control group [122].

The magnitude of the benefit was positively correlated with adherence to the Mediterranean dietary pattern, indicating that the cardioprotective effects depend on the intensity and consistency of dietary change. Mechanistic studies have demonstrated that the key components of this diet—extra-virgin olive oil and nuts—possess potent antioxidant, anti-inflammatory, and vasoprotective properties.

Extra-virgin olive oil is rich in hydroxytyrosol, tyrosol, oleuropein, and secoiridoids, which are polyphenolic compounds that are capable of reducing lipid peroxidation, upregulating endogenous antioxidant enzymes (such as superoxide dismutase, catalase, and glutathione peroxidase), and restoring nitric oxide (NO) bioavailability. Nuts—including walnuts, almonds, and hazelnuts—provide vitamin E, omega-3 fatty acids, and phytosterols, which improve the lipid profile and reduce levels of systemic inflammatory markers such as C-reactive protein (CRP), interleukin-6 (IL-6), and tumor necrosis factor-α (TNF-α) [122].

Thus, the Mediterranean diet not only mitigates classical cardiovascular risk factors but also corrects the underlying redox imbalance through a combined modulation of oxidant and antioxidant systems. This integrated approach explains why the observed cardiovascular benefits surpass those achieved with isolated vitamin supplementation [121,122].

In addition to this, a study was carried out by researchers from the Reina Sofía University Hospital in Cordoba with patients with established coronary heart disease (aged 20–75 years) randomly assigned in a 1:1 ratio by the Andalusian School of Public Health to receive a Mediterranean diet or a low-fat diet intervention, with a follow-up of 7 years. This revealed that in secondary prevention, the Mediterranean diet was also superior to the low-fat diet in preventing major cardiovascular events [123].

### 9.2. Better Pharmacological Approaches: Targeted Therapies for the Redox System

In the past decade, research has progressively advanced toward the development of targeted antioxidant therapies that are capable of modulating the specific molecular pathways involved in cardiovascular oxidative stress (OS). According to the systematic review by Jin and Kang (2024), the most relevant advances focus on molecules that activate the Nrf2–Keap1, SIRT1, and eNOS signaling pathways, or that directly neutralize reactive oxygen species (ROS) within the mitochondrial environment [124].

Activation of the nuclear factor erythroid 2–related factor 2 (Nrf2) represents a fundamental cellular defense mechanism against oxidative stress. Experimental models have shown that Nrf2 activation attenuates inflammation, improves myocardial contractility, and protects against ROS-induced apoptosis. Furthermore, commonly used drugs such as statins and metformin exhibit pleiotropic antioxidant effects through the same mechanism—independent of their primary lipid-lowering and glucose-lowering actions, respectively.

In parallel, innovative compounds such as mitochondria-targeted antioxidants, including MitoQ and elamipretide (SS-31), act directly on the mitochondrial respiratory chain, neutralizing ROS generated at complexes I and IV, restoring mitochondrial membrane potential, and preventing the opening of the mitochondrial permeability transition pore. Early clinical trials with elamipretide have reported improvements in left ventricular function and myocardial perfusion, consolidating this therapeutic strategy as one of the most promising approaches in the field of cardioprotection [122,124].

### 9.3. Conceptual Synthesis: From Isolated Antioxidants to an Integrated Redox Approach

The comparison among antioxidant supplementation trials [121], multifactorial dietary interventions [119], and targeted pharmacological therapies [124,125] reveals a profound conceptual evolution in the understanding of oxidative stress. The current paradigm recognizes that the simple chemical neutralization of free radicals is insufficient to halt vascular damage. Instead, the objective must be to restore redox homeostasis by reinforcing endogenous antioxidant defenses and limiting ROS generation at its primary sources.

This integrated perspective explains why the Mediterranean diet, Nrf2-modulating drugs, and compounds such as N-acetylcysteine or coenzyme Q10 yield more consistent cardiovascular benefits than traditional vitamin-based antioxidants. These interventions act not as passive ROS inhibitors but as dynamic restorers of physiological redox signaling, preserving mitochondrial function, NO-dependent vasodilation, and endothelial integrity [122,125].

Collectively, the current evidence converges on a clear conclusion: the most effective antioxidant strategies are those that coordinate modulation of redox, inflammatory, and metabolic pathways, integrating nutritional, pharmacological, and lifestyle interventions. This comprehensive redox-centered framework provides a coherent pathophysiological basis for the prevention and management of cardiovascular diseases, aligning nutrition, pharmacology, and molecular biology within a unified therapeutic direction.

## 10. Conclusions

Oxidative stress (OS) is a central pathophysiological mechanism underlying endothelial damage and the development of cardiovascular diseases such as atherosclerosis, hypertension, heart failure, and ischemic heart disease.

At the cellular level, major ROS sources—including the mitochondrial respiratory chain, NADPH oxidase, xanthine oxidase, and uncoupled endothelial nitric oxide synthase (eNOS)—contribute to endothelial dysfunction, vascular smooth muscle cell proliferation, and the oxidation of low-density lipoproteins (LDL). The accumulation of oxidized LDL (oxLDL) represents a critical event in atherogenesis, triggering immune activation, foam cell formation, and plaque instability.

Oxidative stress biomarkers have emerged as essential tools to advance cardiovascular precision medicine. Despite their promise, their translation to clinical practice remains limited due to analytical variability, lack of standardized reference ranges, and insufficient methodological harmonization.

Regarding therapeutic strategies, the current evidence indicates that isolated antioxidant supplementation—such as with vitamins C and E or β-carotene—has failed to demonstrate consistent clinical benefits. In contrast, comprehensive dietary patterns that are rich in natural antioxidant compounds, notably the Mediterranean diet, have shown reproducible cardioprotective effects.

Emerging redox-targeted pharmacological therapies—including NADPH oxidase inhibitors, mitochondria-targeted antioxidants (e.g., MitoQ, elamipretide), and agents modulating the Nrf2, AMPK, and eNOS pathways—represent the next frontier in cardiovascular therapeutics. However, their clinical application demands further validation through well-designed trials with standardized endpoints.

Future research must prioritize the standardization and clinical validation of oxidative biomarkers, the development of targeted therapies against specific ROS sources, and the integration of nutritional, pharmacological, and lifestyle-based interventions aimed at restoring redox homeostasis.

Ultimately, restoring redox balance—rather than merely neutralizing free radicals—constitutes the most promising paradigm for effective prevention, diagnosis, and treatment of cardiovascular diseases through an integrated, personalized, and evidence-based redox medicine framework.

The future research priorities should include the following: ① resolving the standardization and validation issues of biomarkers; ② developing precise targeted therapies against specific ROS sources (e.g., NOX inhibitors, mitochondrial antioxidants); and ③ exploring personalized clinical trials based on redox stratification.

## Figures and Tables

**Table 1:** Main oxidative stress biomarkers studied in cardiovascular disease.

Biomarker	Origin/Mechanism	Analytical Methods	Pathophysiological Role in CVD	Limitations	Refs.
Malondialdehyde (MDA)	End product of lipid peroxidation of polyunsaturated fatty acids	Colorimetric assays, ELISA, HPLC	Reflects membrane lipid oxidation; elevated in atherosclerosis and myocardial infarction	Low specificity; influenced by diet and sample handling	[24,83]
4-Hydroxy-2-nonenal (HNE)/Acrolein	Reactive aldehydes from lipid peroxidation of arachidonic and linoleic acids	HPLC, GC-MS	Induces protein and DNA adducts; promotes apoptosis and inflammation	Short half-life; preanalytical instability	[93,96]
F2-Isoprostanes	Non-enzymatic peroxidation of arachidonic acid	GC-MS, LC-MS/MS, ELISA	Considered a reliable in vivo marker of lipid peroxidation; correlated with CVD risk	Requires complex instrumentation; costly	[83,97]
Myeloperoxidase (MPO)	Enzyme released by activated neutrophils during inflammation	ELISA	Catalyzes formation of hypochlorous acid; associated with plaque rupture	Sensitive to preanalytical factors (heparinization, temperature)	[24,83,94]
Oxidized LDL (oxLDL)	LDL particles oxidized by ROS in vascular intima	ELISA using monoclonal antibodies	Key mediator in foam-cell formation and endothelial dysfunction	Lack of assay standardization	[83]
Total Thiols (-SH groups)	Plasma thiol pool (cysteine, homocysteine, albumin)	Spectrophotometry, Ellman’s reagent	Reflects overall redox buffering capacity; decreased in CVD	Non-specific; influenced by nutritional status	[97]
Glutathione (GSH/GSSG ratio)	Intracellular redox pair reflecting antioxidant capacity	HPLC, enzymatic assays	Indicator of systemic oxidative balance and mitochondrial function	Requires rapid processing; unstable in plasma	[90,91]
Antioxidant Enzymes (SOD, GPx, CAT)	Endogenous enzymatic defenses against ROS	Spectrophotometric or ELISA assays	Reflect antioxidant response and compensatory adaptation to OS	Variability among tissues; lack of unified reference values	[24,84,89,96,97,98]
Non-Enzymatic Antioxidants (ascorbate, α-Tocopherol, Zn, Se, Mn)	Dietary or plasma antioxidants	Colorimetric or flame photometry	Complementary markers of antioxidant status; support redox balance	Sensitive to diet and storage conditions	[83]

Abbreviations: OS, oxidative stress; ROS, reactive oxygen species; SOD, superoxide dismutase; GPx, glutathione peroxidase; CAT, catalase; and CVD, cardiovascular disease. This table summarizes the most frequently studied oxidative stress biomarkers in cardiovascular disease, their analytical methods, and their main limitations for clinical application.

## Data Availability

No new data were created or analyzed in this study. Data sharing is not applicable to this article.

## Abbreviations

The following abbreviations are used in this manuscript:

LDL	low-density lipoprotein
OS	oxidative stress
ROS	reactive oxygen species
RNS	reactive nitrogen species
eNOS	endothelial nitric oxide synthase
NF-κB	nuclear factor kappa-light-chain-enhancer of activated B cells
iNOS	inducible nitric oxide synthase
BH_4_	Tetrahydrobiopterine
NADPH	nicotinamide adenine dinucleotide phosphate
NOX	nicotinamide adenine dinucleotide phosphate oxidase
SOD	superoxide dismutase
GPx	Glutathione peroxidase
GSSG	oxidized glutathione
GSH	glutathione
HSPs	heat shock proteins
NO	nitric oxide
ET-1	endothelin-1
Trx	thioredoxin
cGMP	guanosie 3′,5′-cyclic monophosphate
ONOO−	Peroxynitrite
ATP	adenosine triphosphate
mtDNA	mitochondrial DNA
oxLDL	oxidized low-density lipoproteins
PUFA	peroxidation of polyunsaturated fatty acids
CD	conjugated dienes
LOO·	peroxyl radical
MDA	malondialdehyde
HNE	4-hidroxynonenal
IL-4	interleukin-4
IL-1β	interleukin-1β
TNF-α	tumor necrosis factor-α
VCAM-1	vascular cell adhesion molecule 1
ICAM-1	intercellular adhesion molecule 1
HDL	high-density lipoprotein
ABCA1	ATP binding casette
Apo A1	apolipoprotein A1
M1	inflammatory macrophages
ANGII	angiotensin II
AP-1	activator protein-1
MAPK	mitogen-activated protein kinase
DM	diabetes mellitus
AGEs	advanced glycation end-products
ACEIs	angiotensin-converting enzyme inhibitors
RAAS	renin–angiotensin–aldosterone system
ASK1	apoptosis signal-regulating kinase 1
